# Effect of antibiotic infused calcium sulfate/hydroxyapatite (CAS/HA) insets on implant-associated osteitis in a femur fracture model in mice

**DOI:** 10.1371/journal.pone.0213590

**Published:** 2019-03-14

**Authors:** Lisa Oezel, Carina Büren, Armin O. Scholz, Joachim Windolf, Ceylan D. Windolf

**Affiliations:** 1 Department of Trauma- and Hand Surgery Medical Faculty, Heinrich-Heine-University, Duesseldorf Moorenstraße 5, Duesseldorf, Germany; 2 Department of Trauma- and Orthopedic Surgery Helios Medical Faculty, University of Wuppertal/ Witten-Herdecke Heusnerstraße 40, Wuppertal, Germany; Kanazawa University, JAPAN

## Abstract

Cerament (Bonesupport Holding, Lund, Sweden) is a bioresorbable synthetic bone substitute consisting of calcium sulfate and hydroxyapatite which is successfully used as a bone graft in bone defects or in delayed and non-unions after fractures. Besides, calcium sulfate/ hydroxyapatite (CAS/HA) could have, attributed to its composition and osteoinductive properties, have great importance in the treatment of bone infections with critical size defects (CSD). Aim of the study was to evaluate the effects of antibiotic infused CAS/HA on inflammation and bone healing in an implant-associated osteitis mice model. In a standardized murine model, the left femur of 72 BALB/c mice were osteotomized, generating a CSD (2,5 mm) with stabilization through a 6-hole titanium locking plate. Osteitis has been induced through inoculation of *Staphylococcus aureus* (SA) into the fracture gap. To analyze the effect of CAS/HA, following groups were generated with either CAS/HA, CAS/HA with gentamycin (CAS/ HA-G) or CAS/HA with vancomycin (CAS/HA-V) insets placed into the osteotomy. Debridément and lavages were progressed on day 7 and 42 to determine the local bacterial growth and the immune reaction. Fracture healing was quantified on day 7 and 42 by x-ray and bone healing markers from blood samples. Progression of infection was assessed by estimation of colony-forming units (CFU) and immune response was analyzed by determination of Interleukin (IL)– 6 and polymorphonuclear neutrophils (PMN) in lavage samples. Osteitis induced higher IL-6 and PMN-levels in the lavage samples on day 7. Both parameters showed a reduction in all groups on day 42. CAS/HA-V revealed a significant reduction of CFU and PMNs in lavage samples on day 42. A positive effect on bone healing could only be shown in non-infected mice. Whereas, application of mere CAS/HA in infected mice did show tendencies of bone destruction and lysis, independent of impregnation with antibiotics or not. Thus, application of CAS/HA in acute implant-associated infections is not recommended. In non-infectious environments or after infect-convalescence CAS/HA could albeit serve as a suggestive tool in trauma and orthopedic surgery.

## Introduction

Osteitis is described as an infection of the bone with a concomitant inflammation involving the bone marrow and the surrounding tissues [1]. These infections can originate from many different mechanisms, whereby a common cause of osteitis is a bacterial incorporation during or after surgical intervention as well as in case of open fractures caused by trauma [2]. Besides the marked progress in operating standards and amelioration of perioperative measures, especially implant-associated and perioperative infections in trauma and orthopedic surgery still represent significant complications [3]. In literature, the incidence of infection shows a wide range from 1% in primary fracture stabilization up to 55% in the treatment of open fractures [4]. Apart from that the development of an osteitis depends on individual risk factors provided by the patient such as obesity, anemia and diabetes [5]. So overall, the interaction of implants with incorporated bacteria and individual defense capacities lead to a localized infection. Regarding medical device-associated osteitis the most frequent germs are counted to be *Staphylococcus aureus* (SA) and *Staphylococcus epidermidis* [1,6]. SA has developed multiple strategies to escape from the host’s immune defense. Among those, generating a protective biofilm with resistance against systemic antibiotic substances, as well as the invasion of immune host cells present fundamental mechanisms [7]. Moreover, the segregation of cytotoxic molecules, like proteases, together with onward infectious process detains fracture healing [8]. Further, these mechanisms lead to an obstructed activation of the innate immune system which consequently hamper fracture healing, e. g. the activation of polymorphonuclear neutrophils (PMN) represents an important mechanism of bacterial defense [9]. Appropriate treatment with the attempt of eradication of osteitis is necessary to prevent life threatening complications such as sepsis [10]. Osteitis is chronological distinguished into an acute and a chronic type. Time borders are set distinctly, from 4 to 8 weeks describing an acute event and all exceeding that characterizing a chronic process [11]. Both osteitis types are addressed by surgical intervention in terms of radical debridement, lavage and removal of implants as the gold standard of therapy, combined with systemic or oral antibiotic treatment [12]. Detriments of intravenous or oral antibiotic treatment are systemic side effects of antibiotics as well as potentially low effects and concentrations at the local infection site. Furthermore, impairment of local vascularity can decrease the effects of oral or parenteral antibiotic administration [13]. Application of local antibiotics can result in an increase of concentrations at the infection site. Moreover, trauma associated fractures or osteitis itself can often leave large bone defects that do not have self-healing potential and lead to even pronounced infect reactions often requiring stabilization by a bone void filler [14]. The use of autologous bone grafts is often subordinate, first in order to avoid donor site morbidity in general, secondary because most of these accrued defects are big in size leaving this option ineligible [15]. Respectively, the use of bone graft substitutes presents a reasonable option. Properties like biocompatibility, biodegradability as well a positive effect on osteoconduction and osteoinduction have high importance as they allow “one-stage” operative procedures and impel bone healing. Cerament (BoneSupport AG, Lund, Sweden) is a synthetic, bioresorbable bone substitute, composed of 60% calcium sulfate and 40% hydroxyapatite. Hereby, the calcium sulfate is intended to be quickly replaced by newly formed bone and the hydroxyapatite is supposed to act as a template to allow further bone ingrowth [16]. CAS/HA biocomposites are often used as bone graft in delayed- and non-unions after fractures [15]. Besides, several clinical studies have demonstrated the efficacy of antibiotic infused CAS/HA with gentamicin sulfate or vancomycin in the treatment of infected bone defects as well as the successful use as a coating on implants [16–19]. McNally et al. for example, present the effective treatment of chronic osteomyelitis with gentamicin loaded CAS/ HA spacer in patients [19]. Limitations of these studies seem to be that they either describe a primary infect prevention or the treatment of a chronic osteomyelitis and results from these studies in general show a specific risk of bias [20]. Moreover, molecular analysis of bone healing, infection progress and immune response during treatment with CAS/HA has not been fully dissected in these studies. A recent animal study suggested an increased bone formation as well as a decreased rate of detectable infection using CAS/HA impregnated with gentamicin in a rat model of osteomyelitis [21]. This study analyzes the effect of CAS/HA in a plain bone defect model with no critical defect as well as no implanted device. Both criteria, namely the existence of a critical size defect (CSD) as well as the association with an implanted orthopedic device present the most challenging aspects of current septic surgery. Especially the presence of foreign surfaces like implants or prosthesis significantly increases the risk for the development of an infection [22]. Related to that, the implant type as well as the size of the bone defect might additionally influence bone healing or inflammatory processes during osteitis.

Therefore, the aim of this study was to evaluate for the first time the effect of antibiotic impregnated CAS/HA insets on fracture healing, inflammatory processes and local as well as systemic immune response in an implant-associated osteitis caused by SA in a standardized murine femur fracture model with a CSD.

## Material and methods

### Ethical statement

The present animal experiments were approved by the local institutional committee on animal care (“Landesamt für Naturschutz, Umwelt und Verbraucherschutz” of the federal state of North Rhine-Westphalia, Germany—file number: 84–02.04. 2014.A396) and are in line with the European Communities Council Directive (86/609/EEC). Specific effort was made to minimize the number of animals. Reporting of the results of the present study adheres to the “Animals in Research: Reporting in vivo Experiments -criteria” (ARRIVE criteria).

### Animals

72 female wild-type BALB/c-mice were used for the study. The age ranged between 10 to 12 weeks with an average weight of 21 g. Mice were kept in the local animal research institution (animal facility of the Heinrich-Heine-University Düsseldorf, Zentrale Einrichtung für Tierforschung und wissenschaftliche Tierschutzaufgaben, ZETT, Germany) in standard polycarbonate (makrolon type II) cages under a conventional 12 h light–dark cycle (7:00 a.m. / p.m.). Mice had free access to food and water. All animal procedures were carried out in accordance to local and national ethical guidelines. Special training in animal care and handling was provided for the research staff.

Before primary surgery or any lavage as well as before euthanasia, mice were anesthetized by i.p. injection of xylazine (5 mg / kg body weight) and ketamine (100 mg / kg body weight). Besides, after any invasive procedure, a single shot injection of meloxicam (5 mg / kg body weight) was administered.

Additionally, for all mice, a single shot injection of meloxicam (5 mg / kg body weight) was administered every day for the first 5 days after primary surgery to minimize suffering and distress. During the study, animal health and behavior were monitored and assessed every day.

Human endpoints to terminate the experiment were predetermined as: signs of pain for more than three days (non- weight bearing of the operated extremity, no sticking to the cage, no free running and/or moving in the cage), food refusal and weight loss > 20%, unsuccessful fracture stabilization or refracture (axial deviation of the extremity, instability), surgically not controllable wound healing disorders or defects, rectal prolapse, permanently ruffled and unkempt fur, abnormal reaction to a stimulus, permanently closed eyelids, automutilation and immobility with inability of food consumption or fluid intake. Impartial of these endpoints, the experiment was supposed to be discontinued and the concerned animal appropriately euthanized if indicated by reasons of animal protection and welfare.

Once animals reached endpoint criteria, the experiment was immediately disrupted and affected animals were first anesthetized and subsequently euthanized.

### Implant-associated osteitis model

A well-established implant-associated osteitis model in mice was used [23]. Briefly, mice were anesthetized and among sterile conditions a skin incision of 2 cm along the left lateral thigh was done and the fascia as well as the muscles were dissected to expose the femur. Subsequently, a 6-hole titanium locking plate with locking self-tapping micro-screws (MouseFix plate, RISystem, Davos, Switzerland) was applied to the femur. After plate fixation, an osteotomy using a Gigly saw (diam. 0.22 mm) was performed in midshaft of the femur to create a bone defect in terms of a CSD. Mice allocated to an osteitis group, infection was induced by inoculation of the fracture gap with 1μl of SA solution (strain ATCC 29213, averaged 1.35 x 10^5^ colony forming units). After primary operation, mice of all groups were re-anaesthetized 7 and 42 days after primary surgery and a standardized lavage with phosphate buffered saline (250 μl PBS twice) and debridement of infected tissue was performed. Local debridement was implemented with a surgical spoon without involving the periosteum. The lavage fluid was recovered, and PBS added to a total volume of 1 ml. The lavage fluid was further analyzed for the number of SA colony-forming units (CFU), PMN and Interleukin-6 (IL– 6). Parallel to the surgical lavage, blood serum was obtained from the mouse cauda for further analysis of serum bone healing markers: alkaline phosphatase (AP) and amino-terminal propeptide of type I collagen (PINP). On day 42, mice were euthanized by cervical dislocation, blood was gained by cardiac punction and a final lavage was obtained. The timescale of 42 days was determined, as complete bone consolidation was assumed at that time.

### Experimental groups

Mice were allocated to six experimental groups. All mice obtained a lavage and a blood extraction on day 7 and on day 42 before euthanasia. In each group an osteotomy and plate osteosynthesis of the femur was performed. Following groups were formed: Two control groups (12 mice / group), one with an isolated CSD and one with a CSD as well as a CAS/HA spacer without antibiotic augmentation. The other four groups provided the osteitis groups, one with an isolated inoculation of the fracture gap with SA, the other three groups with an inoculation of the fracture gap and additional implantation of CAS/HA, CAS/HA-G or CAS/HA-V insets.

### Calcium sulfate/hydroxyapatite insets

Cerament (Bonesupport Holding, AB, Lund Sweden) is a synthetic, calcium-based bone substitute consisting of 60% calcium sulfate and 40% hydroxyapatite. CAS/HA insets were utilized to assess bone healing as well as immune reactions with regards to the CSD. The insets were generated by using a cylindric template measuring 2x2 mm creating a volume of 0,006283 ml. Either CAS/HA bone void filler itself (CAS/HA) or infused with gentamicin (CAS/HA-G, 0,110 mg gentamycin per cylinder, 17, 5 mg/ml gentamicin) or vancomycin (CAS/HA-V, 0,415 mg vancomycin per cylinder, 66 mg/ml vancomycin) was prepared according to the manufacturers guidelines to generate analogues insets.

### Experimental setup and measured data

Fracture healing was examined by radiographic analysis on day 0, 7 and 42. Progress of wound infection was assessed by the counts of SA in the lavage on day 7 and 42. The local immune response was characterized by measuring the quantification of PMN and IL-6-levels in the lavage. The serum AP and PINP concentrations were evaluated on day 7 and 42.

### Counts of colony-forming units (CFU)

The number of CFU was determined from the lavage on day 7 and 42. 200 μl lavage was serially diluted in PBS and four replicates of 10 μl of each dilution plated on Columbia Agar plates with 5% sheep blood. The plates were hereafter incubated at 37°C and counted for Bacterial colonies after 24 h. Results are delineated as CFU per 1 ml lavage fluid.

### Determination of polymorphonuclear neutrophils (PMN)

The local immune response was characterized by measuring the PMNs in the lavage using flow cytometry (FACSCanto II; BD Biosciences, Heidelberg, Germany). FITC rat anti-mouse Ly-6G and APC rat anti- mouse CD 11b antibodies (BD Pharmingen, Frankfurt, Germany) were used.

### Quantification of Interleukin (IL)-6

IL-6 levels in the lavage samples were specified using a commercially available IL-6 ELISA kit according to the manufacturer’s instructions (R&D Systems, Abingdon, UK). The lower detection limit for IL-6 was 15, 6 pg / ml.

### Radiographic adjudication

Standard anteroposterior radiographic images (MX20 Faxitron, Tucson, Arizona, USA; 40 kV, 16 mA) of the femora were taken under anesthesia on day 0, 7 and 42. The fracture gap size was measured at the plate opposing cortical bone and was classified by using a modified osteitis score [24]: 1 point was considered a healed fracture gap. Decreasing diameters of fracture gaps in terms of callus formation representing fracture healing were rated with 2 points. A constant fracture gap meaning no healing was rated with 3 points, an increasing facture gap in terms of lysis was rated with 4 points and obvious destruction of the femur with 5 points.

### Blood alkaline phosphatase levels (AP)

AP activity was determined in serum. We used an AP Assay Kit measuring the AP activity directly without pretreatment (Abnova, Taipei, Taiwan). This method utilizes p-nitrophenyl phosphate that is hydrolyzed by ALP into a yellow colored product. The rate of the reaction is proportional to the enzyme activity and was measured at a wavelength of 405 nm directly at and 4 minutes after reaction (Victor3, PerkinElmer, Waltham, USA).

### Amino-terminal propeptide of type I collagen (PINP)

PINP concentration in serum was measured by an ELISA assay for human N-terminal propeptide of collagen type I (Cloud-Clone Corp., Katy, USA). Manufactures instructions were followed. Intensity of color was read in a microplate reader (Victor3, PerkinElmer, Waltham, USA) and was depicted inversely proportional to the concentration of PINP in the sample. The standard range was 46, 8 pg/ml.

### Statistical analysis

Statistical analysis was performed using GraphPad Prism5 (GraphPad Software, San Diego, CA). Data was first tested for normality using D´Agostino and Pearson normality test. Further analysis was regarding the distribution of the data accomplished with either two-tailed t-test, Mann–Whitney-test or Wilcoxon test. P-values ≤ 0.05 were considered significant.

## Results

### Baseline data

Surgery was performed on 72 female wild-type BALB/c mice in the age range of 10–12 weeks and a weight range of 18–27 g (mean 21 g). The experimental groups were divided into respectively six groups as described before. Overall, 21 mice died during experimental procedures (mortality rate: 29,17%). 10 of these animals died peri- or postoperative (anesthesia, cardio-pulmonary instability), 9 of these mice were early euthanized as they met endpoint criteria (3 mice because of immobility and non-weight bearing of the operated extremity, 6 mice because of non-controllable wound defects). 2 mice were found dead in the cage with no evident reason for death. 51 mice were considered for analysis.

### Infection progress

**Fig 1:**
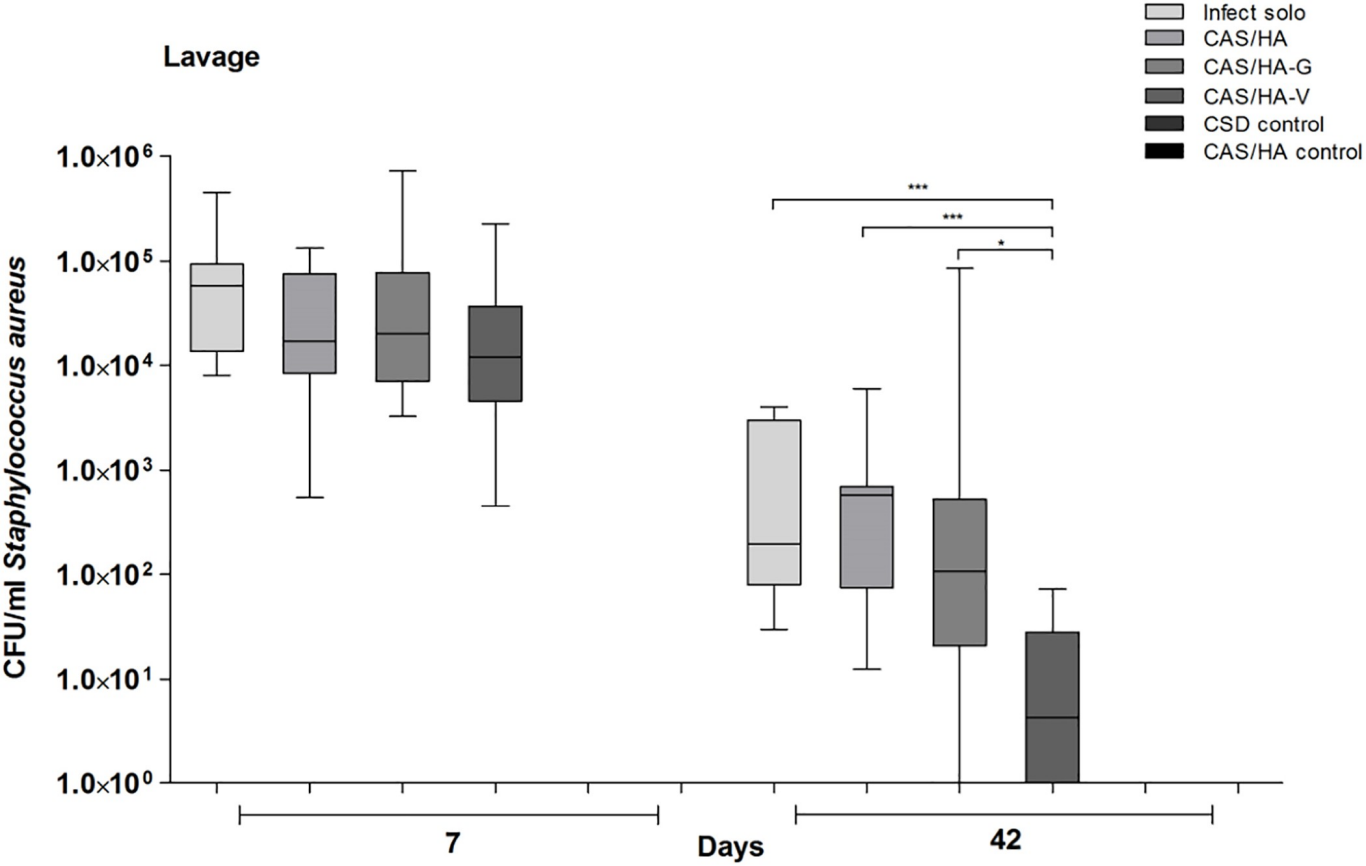
Detection of counts of *Staphylococcus aureus* (SA) around the facture side. Illustration of Colony-forming units (CFU) in the lavage samples obtained on day 7 and 42. On day 42, a significant reduction of CFU in mice with CAS/HA-V could be detected compared to all other experimental groups. * p< 0.05, ** p<0.01, *** p<0.001. Statistical analysis was performed using Mann-Whitney-test.

Infection progress was verified by estimation of the numbers of CFU gained from the lavage on day 7 and 42. Overall, a significant reduction of the local infection could be detected in all groups on day 42 compared to all groups on day 7 (Fig 1). However, mice with CAS/HA-V revealed a significant reduction of detected CFU compared to the other experimental groups on day 42. (Infect solo vs CAS/HA-V: p = 0.0004, CAS/HA vs CAS/HA-V: p = 0.0007, CAS/HA-G vs CAS/HA-V: p = 0.0256). For clearer depiction, data is presented on an exponential scale and therefore control groups, as not being inoculated with bacteria, cannot be represented in bars.

### Inflammatory response

**Fig 2:**
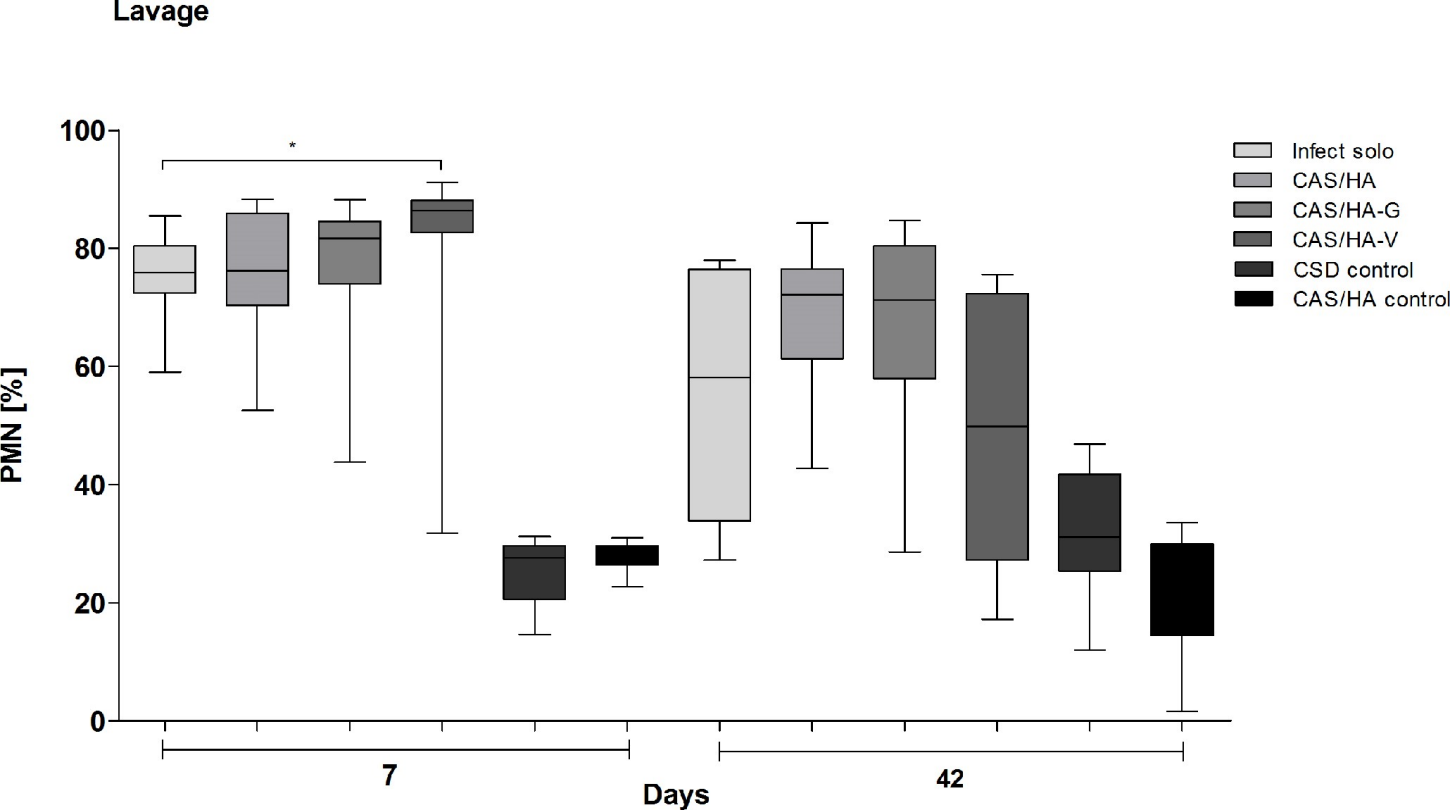
Quantification of polymorphonuclear neutrophils (PMNs). Illustration of PMN- levels in lavage samples on day 7 and 42. Significantly higher counts of PMNs in all experimental groups in comparison to control groups on day 7 and on day 42. On day 7, significantly higher PMNs in mice with CAS/HA-V compared to merely infected mice. * p< 0.05, ** p<0.01, *** p<0.001. Statistical analysis was performed using two- tailed Student’s t-test and Mann-Whitney-test.

The inflammatory response was analyzed by quantification of the PMN and IL-6 levels in lavage. The infection in corresponding groups induced a significant inflammatory response with an increase of local PMNs on day 7 compared to all control groups (p = 0.0002 and p≤ 0,0001) (Fig 2). Moreover, on day 7, significantly increased PMN levels could be detected in mice with CAS/HA-V compared to merely infected mice (p = 0.0115). On day 42, overall a reduction of PMNs in all infectious groups can be detected compared to infectious groups on day 7. Control groups also on day 42 reveal significantly lower concentrations compared to experimental groups. (Infect solo vs CSD control: p = 0.0311 and vs CAS/HA control: p = 0.0021; CAS/HA vs CSD control: p = 0.0007 and vs CAS/HA control: p = 0.0006; CAS/HA-G vs CSD control: p = 0.0002 and vs CAS/HA control: p<0.0001; CAS/HA-V vs CAS/HA control: p<0.0012).

**Fig 3:**
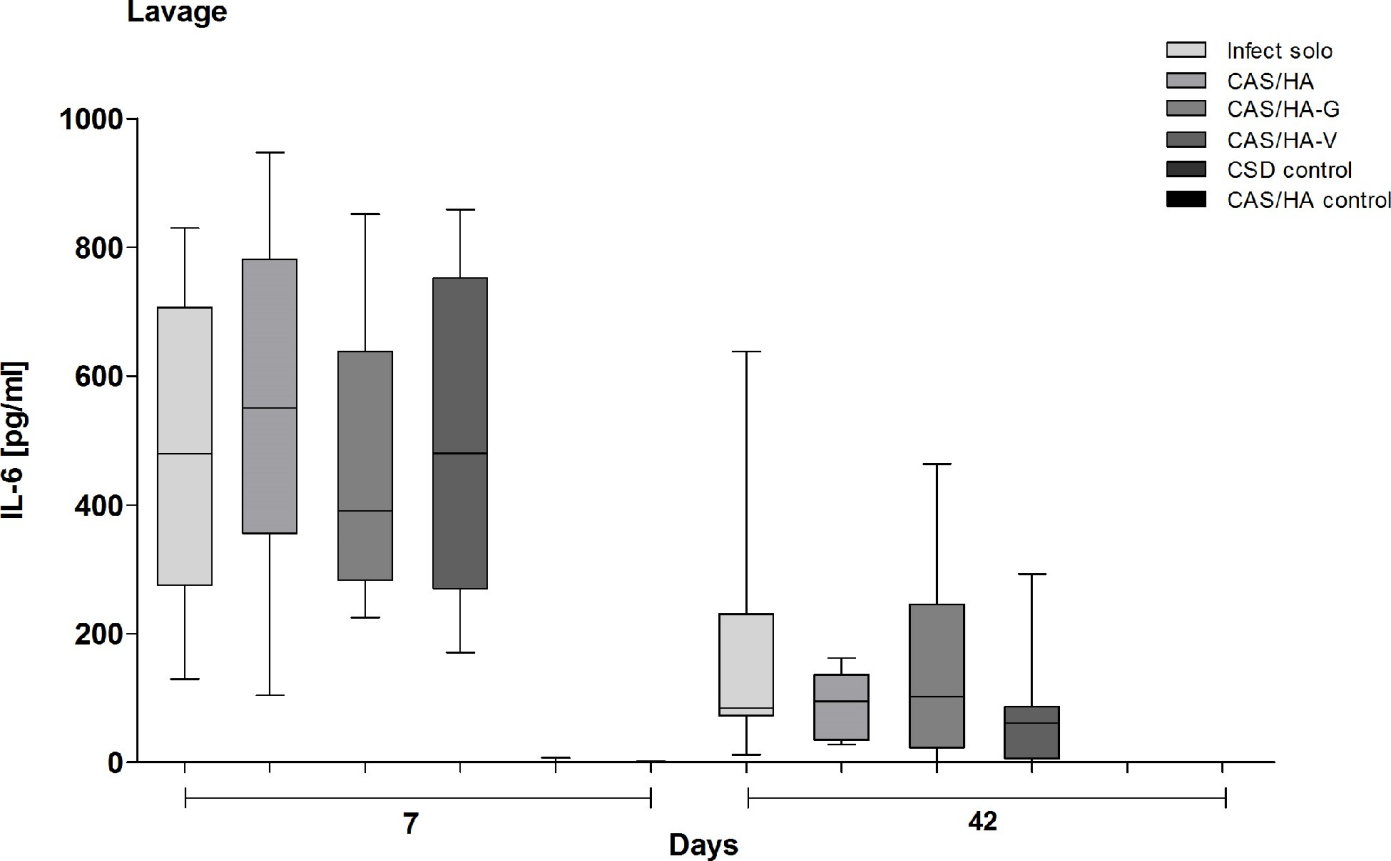
Quantification of Interleukin (IL)– 6 levels. Illustration of IL- 6 levels in lavage samples on day 7 and 42. A reduction of IL-6 levels in all mice groups on day 42 is detectable compared to day 7. All experimental groups on day 7 show significantly higher values of IL-6 compared to control groups (p = 0.0002 and p≤ 0.001). Also, on day 42 control groups show significantly lower IL-6 levels (p = 0.0156 and p = 0.0078). Statistical analysis was performed using two- tailed Student’s t-test, Mann-Whitney-test and Wilcoxon Test.

Also, IL-6 as a marker of systemic infection, shows increased concentrations on day 7 as a response to the local infect constellation compared to day 42 (Fig 3). Additionally, all experimental groups on day 7 revealed significantly higher IL-6 concentrations compared to control groups. (p = 0.0002 and p≤ 0.001). On day 42, merely infected mice vs mice of the CAS/HA control group as well as mice with CAS/HA vs CAS/HA control showed significantly higher IL-6 values (p = 0.0156). Mice with CAS/HA-G vs CSD and CAS/HA control as well as mice with CAS/HA-V vs CSD and CAS/HA control showed a significant increase of IL-6 (p = 0.0156 and 0.0078).

### Fracture healing

**Fig 4:**
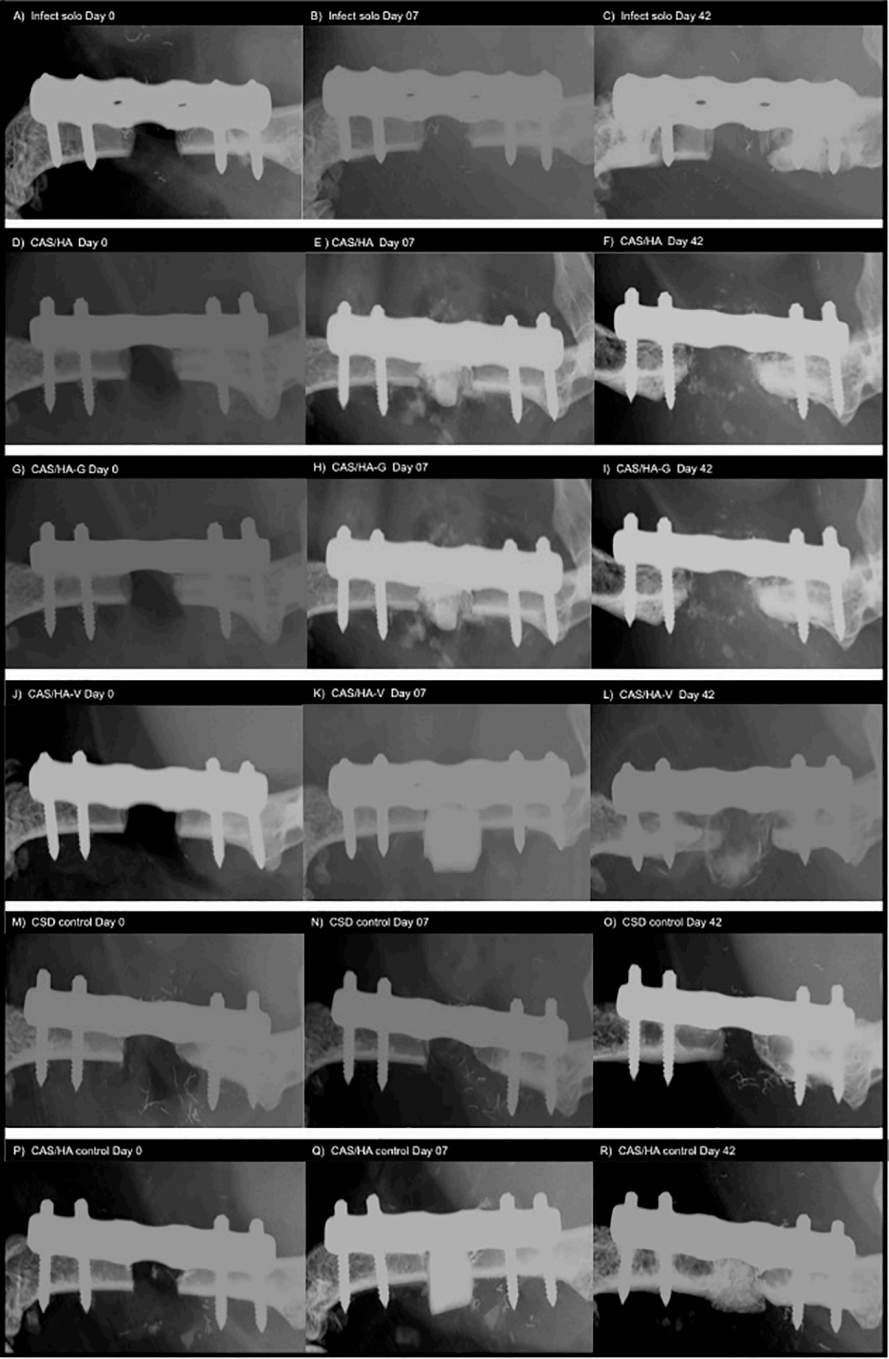
Radiographic analysis of fracture comportment. **A-C)** X-ray scans of the left femur in a solely infected mouse without implantation of a spacer, allocated to the Infect solo cohort after primary surgery, on day 7 and on day 42. The fracture gap shows evidence of destruction and lysis on day 42. **D-F**) X-ray scans of the left femur in an infected mouse allocated to CAS/HA cohort after primary surgery, after implantation of a CAS/HA inset and on day 42. The fracture gap shows a destruction and lysis on day 42 with a dissolution of the CAS/HA inset. **G-J)** X-ray scans of the left femur of a mouse allocated to the CAS-HA-G cohort after primary surgery, on day 7 and on day 42. A dissolution of the CAS/HA-G inset with destruction and lysis of the fracture gap is shown on day 42, despite the use of gentamycin. **J-L)** X-ray scans of the left femur of a mouse allocated to CAS/HA-V cohort after primary surgery, on day 7 and on day 42. Likewise, a beginning destruction and lysis of the fracture gap can be seen on day 42. In contrast to the other groups, a slighter dissolution of the CAS/HA-V inset as well as minor destruction of the fracture gap is visible. **M-O)** X-ray scans of the left femur of a mouse allocated to the CSD control group without infection of the fracture gap and without implantation of a spacer after primary surgery, on day 7 and on day 42. Tendencies of fracture healing with a decreasing fracture gap are visible on day 42. **P-R)** X-ray scans of the left femur of a mouse allocated to the CAS-HA control group without infection of the fracture site, after primary surgery, on day 7 and on day 42. A beginning healing of the fracture gap with integration of the CAS/HA inset are seen.

On days 0, 7 and 42 anteroposterior radiographic images of the femora were taken under anesthesia. All mice with infection did not show a healing fracture gap, independently of the application of CAS/HA insets or antibiotics (Fig 4A–4E). Here, significant results could be shown between infected mice as well as infected mice with CAS/HA and infected mice with CAS/HA-V, as the latter group showed a lysis of the bone whereas the other two even presented a destruction. By contrast, in experimental control groups without infection, a sufficient callus formation and fracture healing in most animals could be detected (Fig 4F).

**Fig 5:**
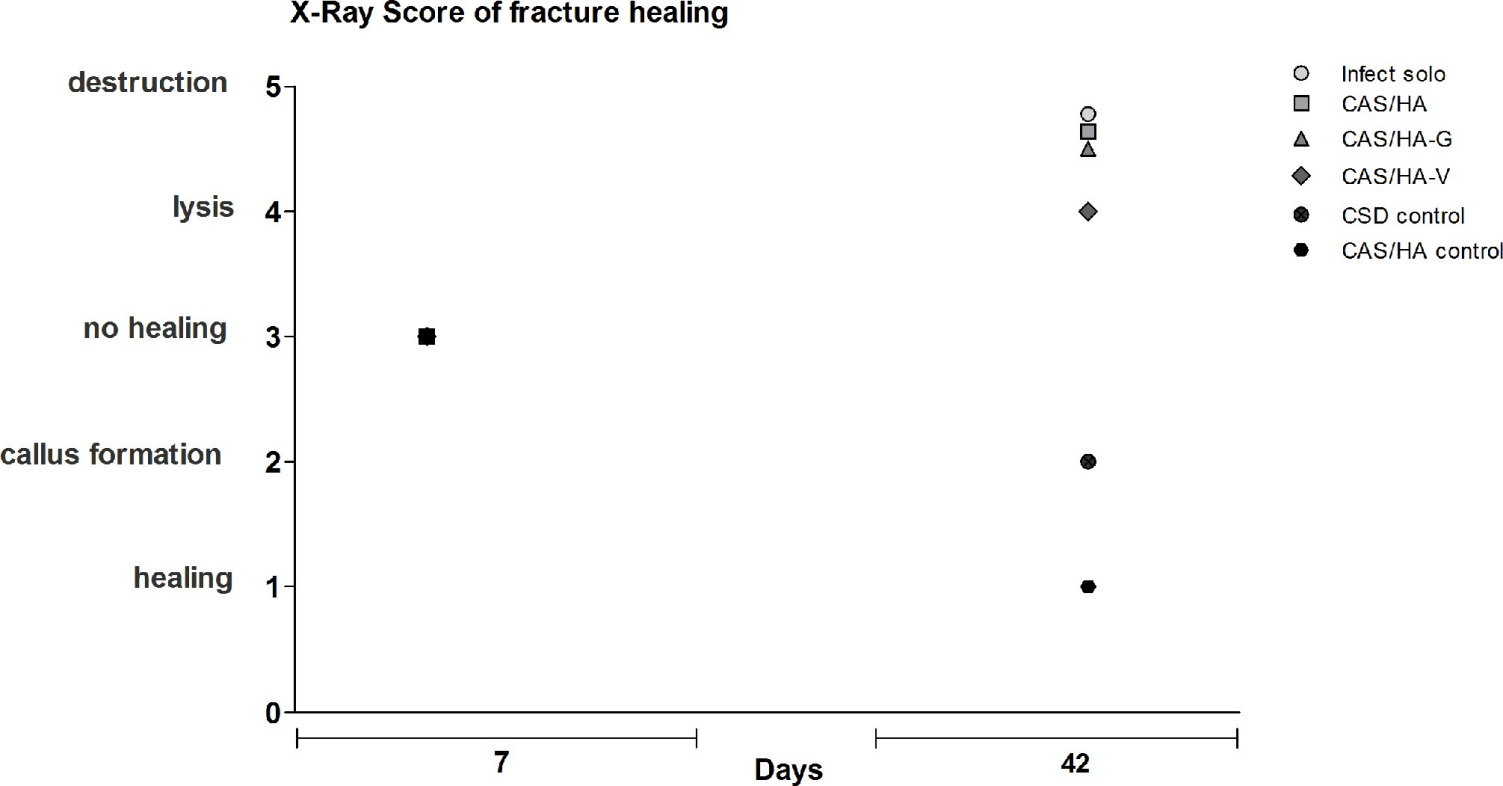
Mean score values of fracture healing. Mean values of the single groups are summarized. A higher frequency of nonunion, lysis and destruction could be shown in all infected mice with or without CAS/HA insets. Bone healing tendencies could only be shown in control groups.

Moreover, fracture healing was evaluated using a modified osteitis score (Fig 5). A higher frequency of nonunion, lysis and destruction could be shown in all infected mice with or without CAS/HA insets. Mice of the control groups showed a fracture healing or callus formation within the observation period of 42 days. This was reflected by mean bone healing scores of 1 (CAS/HA control) and 2 (CSD control), suggesting that CAS/HA supports bone healing in non-infected mice. Mice with infection showed different results. Mice of the groups infect solo, CAS/HA and CAS/HA-G had similar mean values of almost 5 implicating the destruction of the bone. Mice of the CAS/HA-V group suggest that vancomycin infused insets show a less distinct bone destruction, showing a mean score of 4 and therefore an early lysis of the bone.

**Fig 6:**
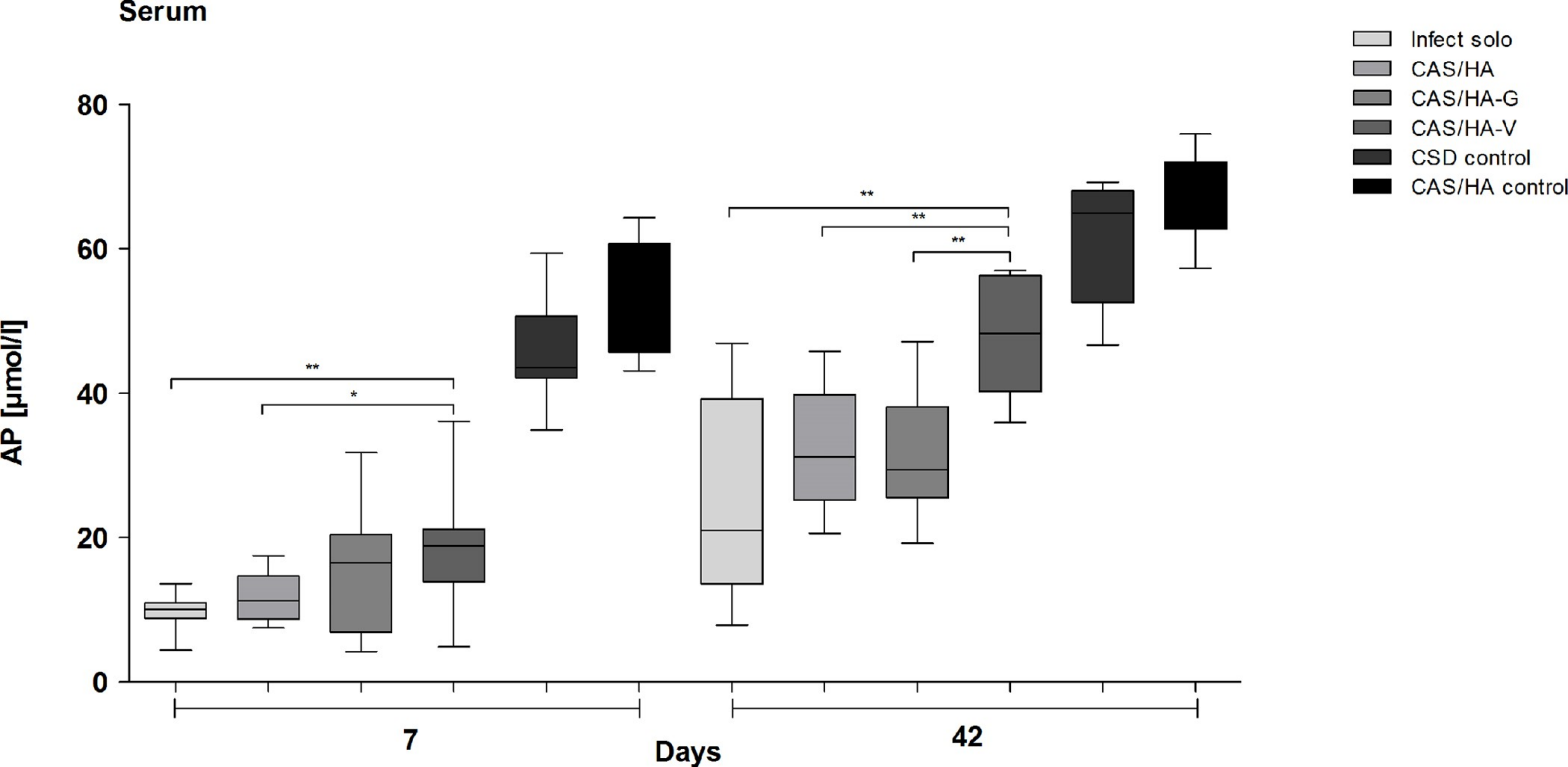
Blood alkaline phosphatase levels. Analysis of alkaline phosphatase (AP) in blood serum revealed significant higher concentrations in all control groups on day 7 and day 42. On day 7, infected mice as well as mice with CAS/HA showed significant lower AP concentrations compared to mice with CAS/HA-V; besides, on day 42, mice with Infect solo, CAS/HA and CAS/HA-G showed significant lower AP concentrations compared to mice with CAS/HA-V * p< 0.05, ** p<0.01, *** p<0.001. Statistical analysis was performed using two- tailed Student’s t-test and Mann-Whitney-test.

Analysis of AP concentration in blood serum revealed that AP- synthesis in general seems to be suppressed during acute infection. On day 42, AP- values raise visibly in all groups compared to AP-values in mice on day 7 (Fig 6). Moreover, on day 7 mice with CAS/HA-V showed significant higher values compared to mice with sole infection (p = 0.0061) and mice with CAS/HA (p = 0.0136). Besides, on day 42 mice with CAS/HA-V showed significant higher values compared to mice with sole infection (p = 0.0037), mice with CAS/HA (p = 0.0062) and mice with CAS/HA-G (p = 0.0037). Moreover, on day 7 as well as on day 42 all mice with set infection, showed significant results compared to both control groups.

**Fig 7:**
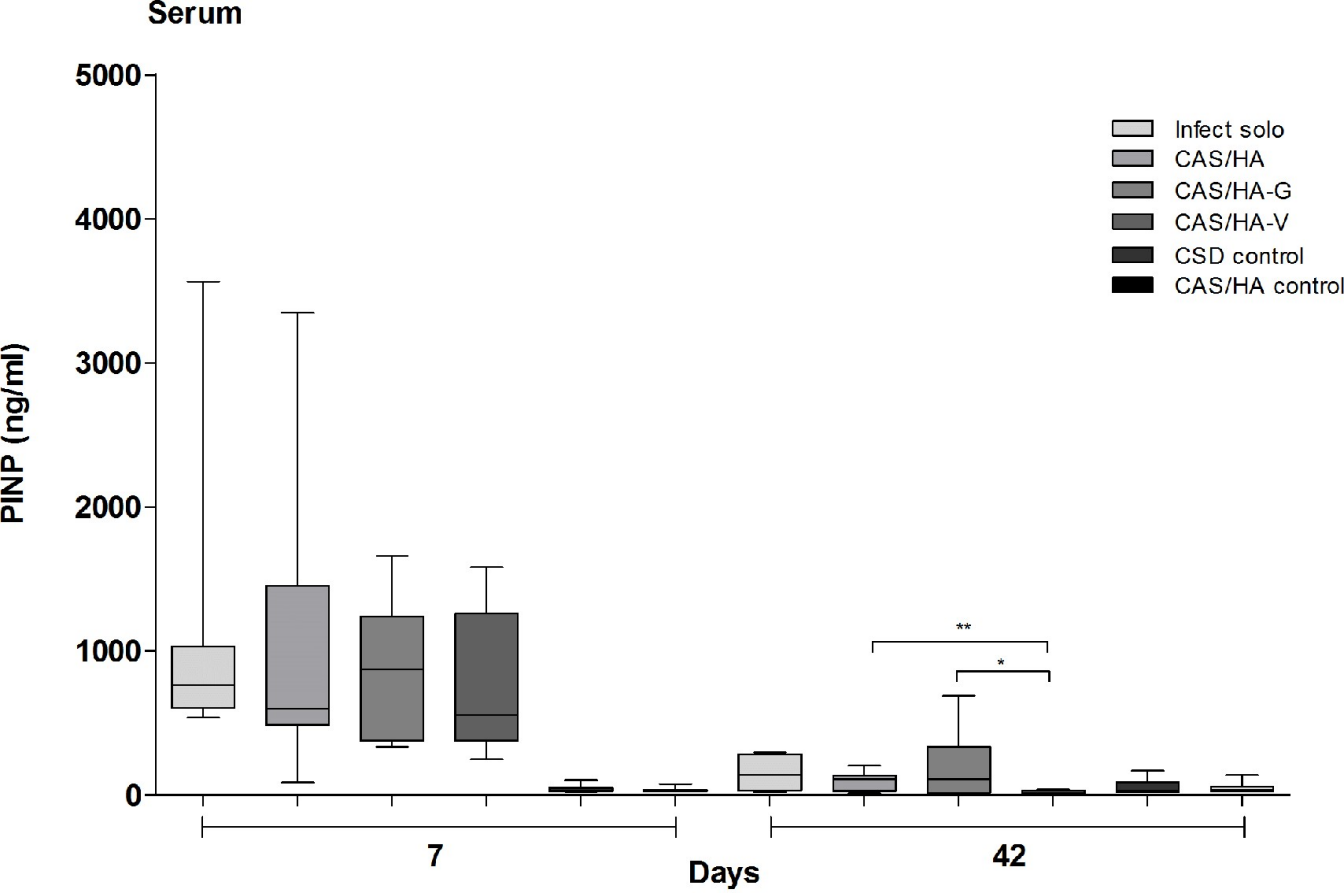
Blood amino-terminal propeptide of type I collagen levels. Analysis of amino-terminal propeptide of type I collagen (PINP) levels in blood samples revealed a significant upregulation of PINP in all experimental groups on day 7 compared to control groups. On day 42, mice with CAS/HA and CAS/HA-G showed significant higher levels of PINP compared to mice with CAS/HA-V. * p< 0.05, ** p<0.01, *** p<0.001. Statistical analysis was performed using two- tailed Student’s t-test and Mann-Whitney-test.

PINP measured in blood serum on day 7 revealed significant higher values in all mice with caused infection compared to both experimental control groups (p = 0.0002 and p ≤ 0.0001). On day 42, infected mice only showed significant higher PINP- values compared to the CSD control group (p<0.0001) as well as mice with CAS/HA-V showed higher values compared to the CAS/HA control (p = 0.0054). In addition, on day 42 PINP- concentrations in mice of the CAS/HA group as well as with mice of the CAS/HA-G group showed significantly raised numbers of PINP- concentrations compared to mice with CAS/HA-V (p = 0.0068 and 0.0356). Overall, PINP- concentrations in infected mice on day 7 showed significantly raised numbers compared to infected mice on day 42 (Fig 7).

## Discussion

Implant-associated infections remain one of the most feared complications in orthopedic and trauma surgery [25]. The current gold standard is radical surgical debridement along with removal of the infected osteosynthesic or prosthetic devices. Besides, initiation of systemic antibiotic therapy as well as application of local antibiotic substances complement the common therapy algorithm. In most cases, osteitis can leave a large, critical defect that requires stabilization and prevent the dispersal or recurrence of infection. Respectively, the use and development of calcium-based bone grafts acting as void fillers that feature bone-building qualities such as autografts and also serve as vehicles for antibiotic substances, is a main target in septic surgery. A systematic review by Hake *et al*. presents a wide range of various synthetic calcium-based bone substitutes either acting as bone grafts or as local antibacterial carriers [26]. Antibiotic-loaded substances serve plenty advantages, as high local concentrations of antibiotics are delivered to the infection site, devoid of all systemic detriments of a vascular therapy [27]. Moreover, bone graft substitutes can provide profitable attributes as biodegradability, osteoconduction as well as osteoinduction [20]. CAS/HA biocomposite is an injectable and moldable bone substitute, which is intended to be transformed to host bone within a few months and likewise serves as a scaffold for further bone remodeling. The use of CAS/HA was previously evaluated in studies, either in terms of a bone graft related to mere bone defects but also with respect to its potential as an antibiotic vehicle in bone infections. The aim of this present experimental study was to evaluate the use of antibiotic impregnated CAS/HA in an implant associated osteitis model to assess its effects on fracture healing, infection progress and inflammatory response on molecular level.

Several studies evaluated the use of CAS/HA in bone defects, emerged from comminuted fractures, osteotomies but also from tumorous events [28–31]. In all these cases, CAS/HA was successfully used, reporting a restored function of the patient’s extremities and both clinical and radiological satisfying outcomes. However, these studies either dealt with non-infectious, healthy bone or the presence of infection was one of the exclusion criteria for the study [31]. Moreover, an experimental work by Zampelis and coworkers conclude that CAS/HA as a coating on implants has no detrimental effects on osteointergration [17]. Another experimental thesis revealed the osteoinductive as well as osteoconductive potential of CAS/HA by presenting its induction of bone in skeletal muscle cells [32]. In this in-vitro study, expression of osteoblastic markers was proved as well as distinctly increased AP- activity as a sign for an early onset of mineralization. Compared to our study, in non-infected mice compatible results could be achieved, as in the x- ray analysis a sufficient fracture healing could be detected. Although, with regards to AP-values, an oppression was demonstrated through the set infection, visibly higher AP concentrations could be detected in experimental groups treated with CAS/HA, particularly in those groups treated with CAS/HA- V. Further, several clinical studies support the use of antibiotic-infused CAS/HA in terms of either infect prevention or addressing the treatment of chronic osteomyelitis using it as a vehicle to operate on the local infection site. Most of these works combine the use of CAS/HA with systemic antibiotic treatment and multiple lavages and debridements. Logoluso *et al*. described in a pilot study the use of CAS/HA impregnated with gentamycin and vancomycin in patients with periprosthetic joint infections as a coating of knee and hip prosthesis [16]. The patients taking part in that study, carried infections from multiple different bacteria, whereby in the majority of cases, infection was induced by *Streptococcus gallolyticus* or *Staphylococcus epidermidis*. All patients underwent a first stage procedure, including removal of all osteosynthetic material with a radical debridement of all infected tissues and the following implantation of an antibiotic loaded spacer. Before implantation of the CAS/HA coated prosthesis, an interval period of 8–12 weeks preceded. Logoluo and collegues described satisfying results with clinical reconvalescence of most patients, postulating that CAS/HA with gentamycin and vancomycin can be used as a coating on implants in septic bone environments. Nonetheless, accessible limitations concerning the further investigation regarding bacterial adhesion and biofilm formation were also shown. McNally and colleagues reported a prospective study about 100 patients with a chronic osteomyelitis, again induced through various germs but mainly by infection through methicillin sensitive SA [19]. All patients were treated with a single-stage protocol, including debridement, removal of implants, stabilization with gentamycin infused CAS/HA, primary skin closure and culture-specific systemic antibiotics for further 6 to 12 weeks. Infect eradication was observed in 96% of the patients at a one year follow up. Another study reports similar results, this time regarding the use of vancomycin impregnated CAS/HA Glombitza *et al*. has reported about a two-stage protocol of a chronic osteomyelitis of the lower limb. Infections were caused by methicillin-resistant SA, multi-resistant *Staphylococcus epidermidis* and polymicrobial, vancomycin-sensitive bacteria [33]. All these clinical studies do not give further insights into molecular processes, comprising different limitations without long-term results. The experimental study by Dvorzhinskiy *et al*. analyzed the effect of gentamycin impregnated CAS/HA in a rat model of osteomyelitis. Osteomyelitis was induced in rats by inoculation of SA into a drill hole. In this work, the author presumes a decreased rate of infection measured by bacterial culture and as a decrease of neutrophil reaction (histology) as well as an increased new bone growth (micro CT-analysis) with the use of CAS/HA with vancomycin compared to the use of mere CAS/HA and omission of bone void filler [21]. Important to mention is that, these results are only said to be valid in debrided and rehabilitated environments. Interestingly the same study also showed that in animals merely treated with CAS/HA, infection rates were higher compared to the groups with gentamycin impregnated CAS/HA and the groups without any bone void filler. These results seem to be partly compatible with the results of our study. We also declare an infect exacerbation in infected mice merely treated with CAS/HA insets, although in contrast to this, in our study also antibiotic impregnated insets did not show any signs of bone growth or regeneration, at least with regards to radiological analyses. Respectively, fracture healing markers like AP-concentrations overall seemed to be suppressed by infection comparing the values on day 7 with day 42, although in mice treated with CAS/HA higher values could be detected, strongest in the group of mice treated with CAS/HA-V. Regarding CFU, again all in all a significant reduction of the local infection could be detected in all groups on day 42 compared to all groups on day 7. In all groups on day 7 as well as in the majority of groups on day 42 stable CFU values were detected. Significant lower values could only be shown among the separate groups on day 42 compared to the group treated with CAS/HA-V, to remain unclear whether the described effect is caused by the impact of CAS/HA itself or rather is a side effect of debridement and lavage. This extends to inflammatory markers such as IL-6 and PMN- levels. Concerning IL-6, as expected lower concentrations in general could be observed on day 42, whereas among the subgroups on both days stable values were detected. Regarding PMN levels, all experimental groups on day 7 show increased PMN levels when treated with CAS/HA or CAS/HA infused with antibiotics, as well as on day 42, except the group treated with CAS/HA-V. It becomes relevant, that for the CFU, PMN and the IL-6 levels on day 42 reductions could only be detected in groups treated with CAS/HA-V, which therefore in this setting intends to show the best results among all other groups. This is partly valid regarding AP- levels as well as radiological analyses, underlining that on the overall no healing tendencies were observed at all, but rather attenuated effects of infect exacerbation compared with the other groups.

All in all, these results can only be partly compared as not only different experimental methods were used, but also crucial differences in the experimental settings are present. On the first, Dvorzhinskiy *et al*. analyzed the effect of CAS/HA in a plain bone defect model with no critical defect, whereas our study analyses processes in an implant-associated osteitis model with a CSD. The CSD created in our study seems to provide a reasonable cause to the process of osteitis, as the effects of the set infection are presumed to be enhanced through the major bone defect. Both criteria are eminent and within the most challenging aspects of current septic surgery [22].

## Limitations

We acknowledge limitations to our present study. First, our study was supposed to consist of a 12- week mice cohort next to the 6- week cohort. Mice of the 12-week cohort were supposed to be euthanized on day 84 to simulate a long-term process of bone consolidation and thus to evaluate and compare results regarding our experimental project.

For the 12 -week cohort, surgery was performed on 72 female wild-type BALB/c mice in the age range of 10–12 weeks and a weight range of 18–27 g (mean 21 g). Surgery was performed using the implant-associated osteitis model in mice as described earlier [23]. Mice allocated to an osteitis group, infection was induced by inoculation of the fracture gap with 2 μl of SA solution (strain ATCC 29213, averaged 2.55 x 10^5^ colony forming units). All following procedures were implemented equivalent to the 6- week mice cohort with the difference of the mentioned bacterial load. Instead of on day 42, in the 12- week mice cohort, on day 84 after primary surgery, mice were euthanized by cervical dislocation, blood was gained by cardiac punction and a final lavage was obtained. Overall, 40 mice died during the experimental procedures (mortality rate of 55,56%). 10 of these animals died peri- or postoperative (anesthesia, cardio-pulmonary instability), 28 of these mice were early euthanized as they met endpoint criteria (3 mice because of immobility and non-weight bearing of the operated extremity, 25 mice because of non-controllable wound defects). 2 mice were found dead in the cage with no evident reason for death.

Obviously, the volume of 2 μl of SA solution was too pronounced and created an overwhelming local infection in mice of the 12-week cohort that constrained premature euthanasia. With regards to the high mortality rate and therefore the small number of animals obtained, results of the 12-week cohort were not stated as representative and could not be analyzed and merely the experimental results of the six-week cohort were considered for analysis.

Secondly, we cannot rule out that CAS/HA might have different reactions or effects regarding other bacteria or poly-microbial infections. In this study, we focused on the most popular pathogen of implant-associated osteitis [34]. Further, the presented model might not necessarily resemble the human condition in every detail, although it shows a significant bacterial load, an impairment of the natural bone healing and an inflammatory reaction, which are important features of the human condition [1]. Finally, we used the term “osteitis” for description of the SA induced bone infection in the present model. A universally accepted definition of the terms “osteomyelitis” and “osteitis” is not yet established [2,3,32,33]. Especially in Anglo-American literature, “osteomyelitis” is the preferred term to describe all kinds of bone infections [2,3]. In contrast, German literature distinguishes both terms “osteitis” and “osteomyelitis” regarding infectious origin and process [11]. Infection progress in osteomyelitis is considered to be caused by hematogenous dissemination of pathogens that firstly affect the bony marrow. By contrast, in osteitis, pathogens intrude from the outside to the inside e.g. in open fractures or in peri-surgery. In the present study, bacterial infection is induced after osteotomy of the femur. Further, the term “osteomyelitis” refers to infection of the bone marrow itself, whereas the term “osteitis” describes an involvement of the entire bone organ, including the bone cortex [35]. Therefore, the present model should resemble the situation of posttraumatic / postsurgical bacterial infections and as bacterial infection involves the bone marrow, the bone cortex and the surrounding tissue, the term “osteitis” was preferred to be used.

## Conclusion

The present osteitis model is sufficient to study fracture healing, infection progress and immune response following an acute implant-associated SA-mediated osteitis in mice. Moreover, this study supports a use of CAS/HA as void filler after infect-convalescence or in non-infectious environments. Application of CAS/HA in acute implant- associated infections, as shown in our model obviously does not lead to limitation of infection and can therefore not be recommended. Nonetheless, the use of CAS/HA in chronic septic environments combined with debridements and if necessary systemic antibiotic therapy, as described in stated studies, could serve as a suggestive tool in trauma and orthopedic surgery.

## Supporting information

S1 File NC3Rs ARRIVE guidelines checklist. A compilation of requirements in reporting in vivo experiments in animals.
(PDF)
